# Understanding who talks about what: comparison between the information treatment in traditional media and online discussions

**DOI:** 10.1038/s41598-023-30367-8

**Published:** 2023-03-07

**Authors:** Hendrik Schawe, Mariano G. Beiró, J. Ignacio Alvarez-Hamelin, Dimitris Kotzinos, Laura Hernández

**Affiliations:** 1 Laboratoire de Physique Théorique et Modélisation, UMR-8089 CNRS, CY Cergy Paris Université, 95300 Paris, France; 2 Universidad de Buenos Aires, Facultad de Ingeniería, Paseo Colón 850, C1063ACV Buenos Aires, Argentina; 3 CONICET, Universidad de Buenos Aires, INTECIN, Paseo Colón 850, C1063ACV Buenos Aires, Argentina; 4 ETIS UMR 8051 CY Cergy Paris Université, ENSEA, CNRS, 95300 Paris, France

**Keywords:** Computational science, Scientific data, Information theory and computation, Statistical physics, thermodynamics and nonlinear dynamics

## Abstract

We study the dynamics of interactions between a traditional medium, the New York Times journal, and its followers in Twitter, using a massive dataset. It consists of the metadata of the articles published by the journal during the first year of the COVID-19 pandemic, and the posts published in Twitter by a large set of followers of the @nytimes account along with those published by a set of followers of several other media of different kind. The dynamics of discussions held in Twitter by exclusive followers of a medium show a strong dependence on the medium they follow: the followers of @FoxNews show the highest similarity to each other and a strong differentiation of interests with the general group. Our results also reveal the difference in the attention payed to U.S. presidential elections by the journal and by its followers, and show that the topic related to the “Black Lives Matter” movement started in Twitter, and was addressed later by the journal.

## Introduction

The debate about the influence of mass media on social opinion has shown peaks of interest each time that a technological breakthrough modified the media ecosystem, mainly by increasing the amount of people that can be reached by broadcasters^1^. The first important one, the invention of the printing press by Gutenberg, has indeed played an important role in the rapid expansion of Calvinism in Europe^2^, although its general influence on the formation of social opinion was mitigated by the fact that most of the population was illiterate. Later, around the beginning of the 20th century, when the wireless radio transmissions appeared and rapidly became a popular entertaining medium, discussions about the foreseeable consequences of the popularization of this new medium were carried in the written press, which by that time had become a traditional one. A review in the New York Times from May 7th 1899 entitled “Future of Wireless Telegraphy” warned: *“All the nations of the earth would be put upon terms of intimacy and men would be stunned by the tremendous volume of news and information that would ceaselessly pour in upon them”*^3^. Needless to say that the same kind of debates took place at the arrival of TV broadcasting^4^.

The rapid growth of digital media certainly triggered again the same kind of discussions but this time, with a major difference: the massive data accumulated on social media platforms allows us to perform measurements about the opinion evolution of large amounts of people. A countless number of articles have addressed different aspects of opinion dynamics based on social networks. A few recent ones are the study of opinion evolution on different selected topics^5,6^, and the characterisation of structural properties of the interaction networks that result from the different functionalities offered by the platforms (like mentions, retweets, follower-friend in Twitter)^7,8^. In particular, there is a recent interest on the formation of *information bubbles* and *echo chambers*—strongly connected clusters of people that communicate only weakly with others^9–11^. Special attention has also been given to the diffusion of rumours and fake news in relation to the COVID-19 pandemic^12^, to the extent that the term *infodemics* was coined to highlight the parallelism with the diffusion of the virus^13–15^.

Nowadays, it seems clear that if media exert an influence on social opinion it is mainly by setting the terms of debate or, in the words of B. Cohen^16^, * the press may not be successful much of the time in telling people what to think, but it is stunningly successful in telling its readers what to think about.* This notion is known as the *Agenda Setting Problem*^17^ and is a long-lasting subject of discussion in Political Sciences, Communication, Social Psychology , Cognitive Sciences, and Media studies. In particular, an open debate concerns the relationship between the notion of *issues* –the subjects that are addressed– and that of *frames* –the attributes assigned to these subjects when they are addressed–^18–20^.

In this work we investigate the agenda setting problem, by studying the dynamics of the different *topics* treated by a traditional medium, *The New York Times* (NYT) journal, and their relationship with the dynamics of the public discussions that take place in Twitter among its followers. Here, the term *topic* designates the subjects treated in both media without attempting to differentiate between issues and frames. This is the standard meaning given in textual corpora analysis which has also been used to address the agenda setting problem^21,22^.

We center our study in the first year of the COVID-19 pandemics, which by its very nature can be expected to become an important driver of public attention. Several works studied the evolution of the opinion in Twitter (and other platforms) during this period, mainly focusing on discussions directly related to health issues, or public policies related with them^23–25^. Here, on the contrary, we aim at understanding how the different topics that interested the society during this period were addressed both by the media and by the public that is in direct relation to them, without assuming a priory the existence of any influence on either direction.

While some recent studies have compared how traditional media and social networks treat *a particular topic* of discussion^26–30^, in this work we search for global patterns characterizing each of them. We have collected a large amount of tweets corresponding to a randomized sample of the over 46M followers of the New York Times (NYT) official Twitter account (@nytimes), during the first year of the pandemic, along with the metadata of the articles published by the journal during that period. This sampling guarantees that we are reaching the topics discussed by users that have expressed an interest in that journal by following its Twitter account. In order to compare with the behaviour of the followers of different media, we have also collected a sample of the tweets published by the followers of other important media of different kinds: written press, radio, television, press agencies.

With this data, we build a semantic network representative of the discussion taking place in social media, based in the co-occurrence of *hashtags* -tagging words starting with the symbol “#”-, chosen by Twitter users. By community detection on this semantic network, we identify the topics of interest discussed in the platform. Additionally, the keywords chosen by the journalists to tag their articles allow us to identify the topics treated by the journal.

In the general context of agenda-setting, the extracted topics from a text corpus might operationalize either frames or social issues, depending on each specific context or dataset, as suggested by different works that use this approach: Danner *et al.* specifically study the correlation between media and public agendas related to organic food, assuming that each extracted topic is a sub-issue inside the general ‘organic food’ issue^31^; Albanese *et al.* perform non-negative matrix factorization (NMF) of the document-term matrix to study the coverage of different issues during the 2016 US presidential campaign^32^; Barberá *et al.* use LDA to study the issues discussed by members of Congress, ordinary citizens, and media outlets on Twitter, a-priori assuming that the extracted topics represent issues^33^.

With a different approach, topic detection has also been used to identify frames: Blei *et al.* use LDA to analyze 8000 articles about the US government support for arts between 1986 and 1997, assuming that “when applied to corpora that cover specific issue domains (like government funding for the arts), topic modeling has some decisive advantages for rendering operational the idea of frame in media research”^34^; Walter and Ophir apply a two-step approach based on LDA and community detection to extract frames on three domain-specific corpus^35^. This differs from our case study, where we are not interested in a specific subject but we investigate the whole news’ treatment during a period.

Our general aim is to look for differences in patterns of information treatment between traditional media and social media. This will be done by analyzing global measures such as issue salience, attention diversity, rank diversity, and reaction times.

By an extensive analysis of these data we aim at getting an insight into the following questions:

- Who talks about what? Do people that follow a journal talk about the same subjects that are published in the journal? In that case, is it possible to quantify to what extent?
- How the attention that the followers of the journal pay to different topics compares to the attention that followers of other media pay to those topics?
- Can we observe any evidence about the agenda setting problem? If so, in which sense?

As discussed by Scheufele et al., agenda setting is an inherently causal theory, however, the research designs and statistical methods employed to study it are seldom suited to make causal inferences^36^. There is no mystery for this, as determining causal inferences in a non stationary time series of events is a difficult problem and we will not address it here. However, one can ask whether the online discussions of the followers of a media are similar, in general and in its temporal evolution, to the salience of the different subjects as treated by the media itself. Moreover, we show that the comparison of the time evolution of the treatment of information in a top-down media like the NYT with the discussions of its followers occurring on line allows us to detect if the salience of a given subject in the NYT precedes or not its follower’s discussions about it.

## Results

We collected data for over a year, starting on January 2020, before the outbreak of the pandemic, from followers of the @nytimes in Twitter, and also from Twitter users who follow Twitter accounts of other media, like @washingtonpost, @WSJ (The Wall Street Journal), @TIME, continuous information television channels like @FoxNews, or @CNN, and also press agencies like @AP (Associated Press) and @Reuters. During the same period, we have also collected the metadata of the publications of the NYT journal, in particular the articles’ headers (see “Methods”).

In order to automatically determine the topics of discussion in Twitter, we build a hashtag network where two hashtags are connected if they appear in the same tweet (see “Methods”). This link is weighted by the number of *different* users that used that pair of hashtags, which diminishes the potential influence of automated accounts.

This semantic network relies on a single assumption: if two hashtags appear in the same tweet, they are likely to refer to the same subject. As a given subject may be addressed to by different hashtags, the topics of discussion in the platform are automatically obtained by community detection in the semantic network^37,38^, and we consider that each community constitutes a topic of discussion in the platform (see “Methods”). The topics treated in the NYT articles are labelled by the keywords given by the journalists to characterize each article.

### Topic dynamics

The entropy of the vocabulary (hashtags for Twitter and keywords for the NYT journal) allows for a global comparison of the dynamics of the discussion in both media (see “Methods”). Entropy is a physical quantity widely used in Statistical Physics to characterize the width of a probability distribution, and therefore, the information obtained by the observation of a given event of such distribution. This notion is useful in different disciplinary fields and has already been used to capture attention diversity in previous agenda setting studies^21,38^. Low values of entropy indicate that the discussion is concentrated around few hashtags or that the information in the journal can be tagged by a few keywords, while high values reveal that a variety of hashtags or keywords are being used.

**Figure 1:**
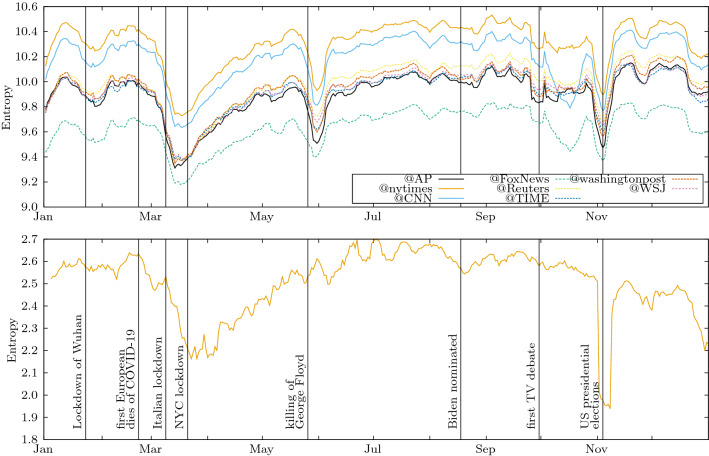
Entropy of the vocabulary as a function of time. Top panel: Time variation of the hashtags’ distribution entropy corresponding to the frequencies of usages of each hashtag by the Twitter followers of the accounts of the media listed in Table 3. Bottom panel: Time variation of the keywords’ distribution entropy corresponding to the keyword usages in the articles of the New York Times. The vertical lines indicate as a reference, the time location of important events during the studied period. In both cases the computation has been done daily with a 7 day rolling averaging (3 days before, 3 days after) to remove the effect of weekdays.

Figure 1 (top) shows the temporal evolution of the entropy of the hashtags used by the followers of different media. The dates of important events are marked as temporal references. As the entropy is an extensive variable, it is not surprising to see that the corresponding values are, in general, larger for Twitter than for the NYT, since the number of hashtags is much larger than the number of keywords.

Also, the entropy curves corresponding to the @nytimes and @CNN followers, who are significantly more numerous than those following the remaining media accounts, are globally larger than the other curves, as more users naturally lead to more hashtag usages. The unexpected, opposed situation is observed for the @FoxNews followers, whose entropy is always the lowest in spite of the fact that this is not the smallest group, revealing that @FoxNews followers use fewer hashtags than expected by their number. This is not due to a particular event that could have interested these followers, but it is constant in time, which indicates a characteristic of those users.

Figure 1 (bottom) shows a different dynamics for the evolution of the entropy of keywords of the NYT journal. Although the publications in both supports are naturally attached to real life events, a detailed inspection of the most popular topics in both media confirms also structural differences. For instance, the discussions about the ‘Black Lives Matter movement’ notably give an earlier signal in the entropy of Twitter, while its influence is hardly detectable after the killing of George Floyd in the entropy of the NYT keywords. This observation may be related to the fact that, unlike Twitter, a journal follows editorial policies which mostly lead to a balanced reporting about different topics.

As expected, the ”Coronavirus” topic dominated both online discussions and also the journal articles, capturing the attention during a long period, as shown by the wide entropy decrease in March-April. As COVID-19 influences most aspects of life, it appears in many sections of the journal and has considerable influence on the entropy.

The ‘Presidential Elections’ topic, visible in both entropy panels, shows a steeper valley for the NYT curve (deeper than the dominating ”Coronavirus” topic). This reflects the importance of the covering of elections by the journal, as NYT publishes, among others, one article of the election results for each of about 400 districts of the United States, really focusing on the subject during this period.

**Figure 2:**
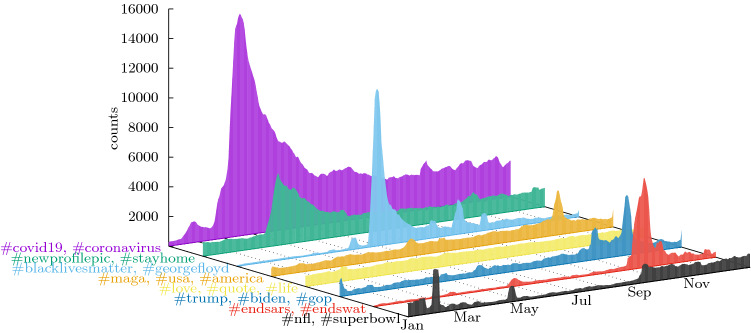
Dynamics of the eight largest topics of the discussion in Twitter by the followers of @nytimes account. The topics are identified in the semantic network of co-occurring hashtags by Infomap (one level down, i.e., path length of 2). They are labelled by the most used hashtags belonging to each topic. The vertical axis shows the number of unique users using a hashtag belonging to the community of the corresponding topic on a given day, smoothed with a rolling average over seven days to eliminate the cycles introduced by weekends.

A closer look at the topics’ evolution allows us to confirm that the remarkable decrease observed in the entropy curves around the dates of important social events are indeed caused by topics related to them.

Figure 2 shows the evolution of the eight most popular topics which are labelled by the most used hashtags in the corresponding community. We can also detect the effect that the pandemic had on the public discussion about subjects that seem a priori completely unrelated to it. For example, the topic labelled by the hashtag #newprofilepic, includes other hashtags related to locations and also the hashtag ‘#flashbackfriday’ which is used to tag pictures. We find that this topic becomes connected to the coronavirus pandemic via the #stayhome hashtag, presumably because of the changing nature of pictures posted under these hashtags due to lock-down period.

The fact that our method lets the topics emerge, instead of following a set of hashtags or keywords chosen a priori, reveals interesting facts. We find a topic whose popularity may appear as surprising in US society, labelled by the ‘#endsars’ hashtag. In fact, this topic refers to the demonstrations against police violence sparked by videos showing brutality of the Nigerian police organization SARS (Special Anti-Robbery Squad, not to be confused with the SARS-Coronavirus). After detecting the users who talked about this subject we found that most of their accounts were tagged abroad (see Supplementary Material).

**Figure 3:**
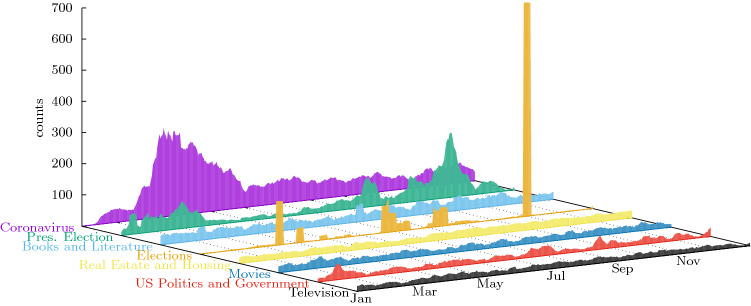
Dynamics for the 8 most used primary keywords tagging the NYT articles (shortened names used in the labels). The vertical axis shows the number of primary keyword usages on each day, smoothed with a rolling average over 7 days to eliminate structure introduced by weekends (see Supplementary Material).

The dynamics of the topics treated by the NYT journal, is shown in Fig. 3. As for Twitter, we also observe topics reporting events, like ‘Presidential Election’ or ‘Coronavirus’ and those which correspond to regular reporting of different aspects of social life like ‘Books and Literature’. As signaled by the entropy curves, the ‘Black lives matters’ topic which dominates in Twitter (cf. Fig. 2) is not even among the leader keywords depicted here. On the contrary, the ‘Presidential Election’ and ‘Elections’ keywords, which also includes articles about the results of the presidential election for all districts, show that this subject dominates the journal attention even in the background of the pandemics, while it is much less important for its followers which refer to it by the topics labelled by #maga and #trump.

### Rank diversity

The rank-diversity measure was introduced to study the evolution of languages, which takes place over long periods,^39^ by analysing the evolution of the rank of single words or n-grams^40^. By definition (see “Methods”), it has a low value when few hashtags or keywords have occupied the observed rank during the corresponding period. For the first ranks, this low value reflects that the leading subjects were represented by few hashtags or keywords.

**Figure 4:**
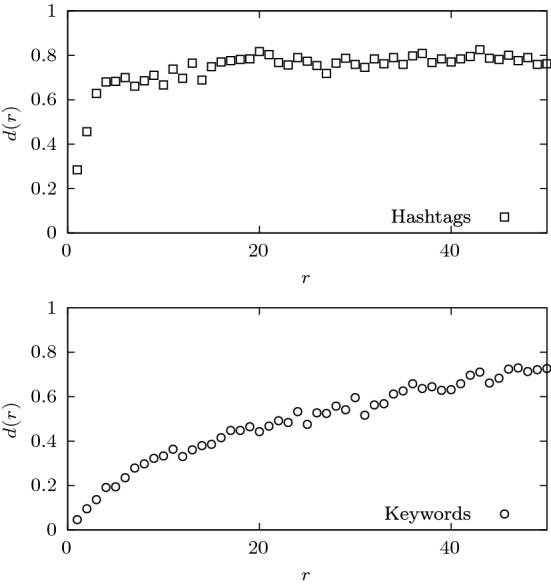
Daily rank diversity *d*(*r*) for the 50 most used hashtags (top panel) and keywords (bottom panel).

The plots of Fig. 4,which concern a much shorter time-scale, differ from the sigmoid curves characteristic of language evolution. In Twitter and in this particular period, one could have expected to have first ranks completely dominated the few variations of COVID-19 hashtags. However, this is not exactly the case. Only rank one and two have a diversity below $d(r) < 0.5$ (with $d(1) \approx 0.3$ and $d(2) \approx 0.45$), while the remaining ones have $d(r) > 0.5$. This implies that even the first ranks are occupied by many different hashtags (notice that $d(r) = 0.5$ corresponds to 180 different hashtags in position *r*; see “Methods”). This doesn’t necessarily mean that the users were ignoring the pandemic in their discussions but, as it affected very different aspects of society, it can be addressed to by many different hashtags. In fact, we have found that the COVID-19 topic is composed of about 700 hashtags (see Supplementary Material), several of which are very popular and contribute to the relative variability of the first ranks.

The rank diversity clearly captures the structural difference between these two media, showing a completely different shape for the keywords of the NYT journal. Since the keywords are curated, the bottom panel of Fig. 4 reveals that dominant topics of the journal are addressed by very few keywords. This shows that the NYT has a narrower focus on a selected group of topics than their followers on Twitter.

### Reaction of the followers of NYT to its publications

The measurement of reposting latency between the initial issue of a piece of news in a media and the reaction of the receivers has been studied in several contexts as a proxy of different aspects of social behaviour. The interpretations of the measured lag depend on the context of the study, for example it may relate to cognitive processing speed, associative strength in memory, and spontaneous cognitive formation of a construct^41^, but it can also reveal properties of the media where the information is diffused like email and online forums^42^, or social media^43,44^. Reaction times have also been related to the *public issue attention*, given that the public has a limited capacity to follow the different debates that take place in the public arena where an issue chases another^45,46^.In the context of agenda setting theory, shorter/longer reaction times have been associated to cognitive congruence/dissonance, giving rise to the agenda melding process^47^; also, a very recent statistical study of a sample of Twitter users showed that multiple issues could distract user’s attention, thus leading to the low reposting speed^48^.

Here we investigate the patterns that characterize the reactions of the followers of the @nytimes account to the articles and tweets published by the journal. We observe two different kinds of reaction: a *direct* one takes place directly on the Twitter platform, when the followers retweet, quote or reply to the tweets published by the journal’s account. An *indirect* reaction, instead, takes place by the means of the ‘share on Twitter’ button of the website nytimes.com where the followers of the journal can tweet a link to the article of their choice. These two kinds of interaction between the journal and its followers are characterized by different regular patterns.

**Figure 5:**
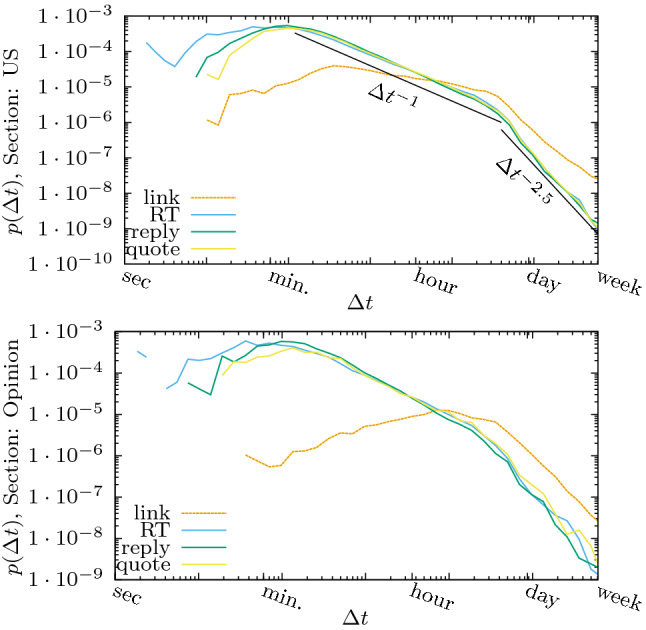
Distribution of the delay times, $\Delta t$: Elapsed time between the direct reaction of the users (retweet, reply or quote) and the issue of a tweet from the @nytimes account or between the indirect reaction, retweet of an the article via the “share” button in the website, and the appearing of the article in the journal (violet lines, labeled *link* in the figure.) We show here the distributions corresponding to two sections of the journal in which the corresponding articles appeared. Top: “U.S.” section Bottom: “Opinion” section (for other sections, see Supplementary Material).

Figure 5 shows the distribution of *reaction times*, $\Delta t$, the delay between either the tweet of the @nytimes account and the direct reaction of the user, or the delay between the publication of an article online and the tweet published by the follower using the website button. The first observation is the very broad range spanned by the reaction times, going from seconds to a week. There is a striking difference in the shape of the curves corresponding to direct reactions, where the distribution of reaction delays seem to fit a power-law with a breaking of the slope around 10 hours, and that of indirect reactions which are much slower and start in general by a very broad shallow peak followed by a power law decrease again around 10 hours.

The numerous retweets happening within the first second of the original tweet, suggest the presence of automated users. The qualitative behaviour of the reaction times is the same for the three direct reactions curves. Fitting a power law after the maxima of the distributions, we find a breaking of the slope from $\Delta t^{-1}$ to a fastest decrease, $\Delta t^{-2.5}$.

The extremely similar behavior of all direct interactions suggests that this process may strongly be influenced by the way in which the platform presents the tweets of followed users, where older tweets are pushed out quickly from a user’s timeline by newer tweets. In this case, the first retweets of an article may trigger more retweets from the users that might have lost the original tweet from @nytimes in their timeline, in a manner of a self exciting process^49,50^.

The longer reaction times observed for indirect reactions are expected, assuming that followers which are on the NYT website are more likely to read the article before sharing it, such that the most probable reaction time is shifted to multiple minutes or hours. Also here, we observe a strong decrease at the $\approx 10$ hour mark. An important difference with the direct reactions is that the distribution of response times for link sharing does not look universal, showing a different shape for different sections (see Supplementary Material).

Despite the extremely similar shape of the delay distributions of the direct Twitter interactions, the median delay time fluctuates by more than a factor of two for different sections, as shown in Table 1. Links to articles about books and art are posted for longer time (median delay of over one day), than national (U.S.) or international (World) news (median delay of about half a day), which seems expected considering that book reviews should remain of interest for longer times than the typical everyday news item. However, the behaviour of the direct reactions shows the opposite tendency: Books and Art are the sections with the shortest median delays before retweets, while national and international news are amongst the slowest sections regarding retweets. We remark that we only consider those tweets that reply directly to the original tweet of @nytimes and not replies to other replies. Therefore, the shorter median delay observed for replies is not caused by fast back and forth discussions.

**Table 1:** Median of the delay $\Delta t$ in minutes for different sections and different types of interactions shown for the eleven largest sections sorted by decreasing number of articles assigned to the sections.

Section	${\Delta t_{\textrm{RT}}}$	${\Delta t_{\textrm{reply}}}$	${\Delta t_{\textrm{quote}}}$	${\Delta t_{\textrm{link}}}$
U.S.	81.9(5)	52.3(6)	71.0(9)	643(3)
World	90.5(9)	45.9(7)	73.4(17)	827(6)
Opinion	68(3)	38(2)	83(7)	952(4)
Arts	53(2)	37(2)	46(2)	1526(18)
Business Day	75(1)	49(1)	89(4)	814(7)
Sports	42(2)	27(2)	44(4)	713(15)
New York	85(1)	45(1)	73(2)	597(6)
Books	45(2)	36(3)	40(5)	1646(28)
Style	103(5)	50(3)	107(7)	1065(19)
Movies	53(3)	34(2)	52(4)	1259(35)
Real Estate	29(4)	40(7)	62(20)	1735(38)
All articles	76.7(3)	47.0(3)	67.8(5)	858(2)

Despite the shape of the distribution being very similar (see Supplementary Material), the median delay fluctuates by more than a factor of two depending on the section. The number in parentheses specifies the standard error in units of the least significant digit, obtained via bootstrap resampling^51^.

### Characterization of the users

The dynamical study of the discussion taking place in Twitter during the considered period shows that some groups of users synchronize in phase or in anti-phase at some particular moments, revealing that most of them are discussing about the same subject or talking about completely different ones, respectively.

A *dynamical topic vector*, whose dimension is equal to the total number of detected topics, is associated to each user. Each component of this vector indicates whether the corresponding user has tweeted more or less than the average population about the corresponding topic, as a function of time (see “Methods”). As we have determined the communities (topics) in a semantic network that includes the tweets of the followers of all considered media, this procedure ensures that we do not miss topics which might be of scarce importance for followers of @nytimes, but relevant for followers of other media.

**Figure 6:**
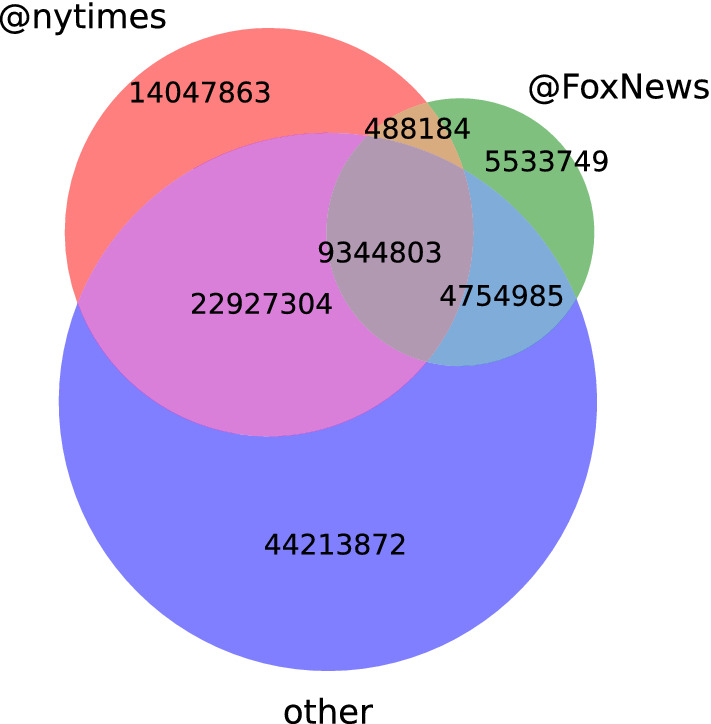
Venn diagram of “followship” relations showing the intersection among the followers of @nytimes (pink) and @FoxNews (green) and the other media (blue).

Users are divided into groups according to the media they are following, among the 10 most followed media in US, listed in Table 3 (see “Methods”). Figure 6 shows that many users follow more than a single medium. Users following a single medium are called *exclusive followers*. Most of the media considered here hold a neutral or liberal position on the political spectrum with a similar entropy of their vocabulary, as shown in Fig. 1; the exception being @FoxNews, which is considered politically conservative and whose entropy is the lowest as discussed in subsection ‘Topic dynamics’.

**Figure 7:**
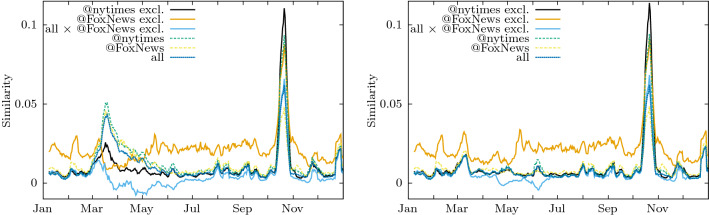
Dynamics of self and cross-similarities corresponding to sub-populations that follow different media accounts in Twitter. For clarity we concentrate on the curves involving the followers of @nytimes and @FoxNews, along with a randomized sample that includes followers of all media, labelled “all” (more curves in the Supplementary Material). The labels ‘@nytimes excl.’ and ‘@FoxNews excl.’ refer to the sub-populations whose members only follow the cited medium. ‘all $\times$ @FoxNews excl.’ is the cross similarity between the exclusive followers of @FoxNews in our dataset and all users (including the followers of @FoxNews) in our dataset. Top panel: Self-similarities of the different sub-populations along with the cross-similarity of exclusive followers of @FoxNews against the set of all users. Bottom panel: Same data recomputed after the suppression of the #covid topic from the topic vectors.

Users in each media group are compared by measuring how similar their dynamical topic vectors are at a point in time; this self similarity measure is described in the “Methods”. The top panel of Fig. 7 shows two remarkable peaks in the self similarity curves, one by the end of March 2020 which corresponds to New York city’s lockdown and the other by the end of October 2020, the exception being the self similarity of the curve of exclusive followers of @FoxNews, which has only one. The bottom panel, shows the self-similarity recomputed suppressing the “COVID” topic from the topic vectors and the disappearing of the peak of March 2020 confirms that the synchronization of the discussion corresponds to this event.

Due to the large overlap of followers of different media, illustrated in Fig. 6, it is not surprising that the self-similarity curves of non-exclusive followers of different media show a qualitatively similar behaviour. However, scrutinizing the exclusive followers of @nytimes and the exclusive followers of @FoxNews we observe they behave differently. When the “COVID” component has been removed from the topic vectors of the users, the self-similarity of the exclusive followers of @FoxNews is higher than that of the rest of the users (including that of the exclusive followers of @nytimes), except for the large peak at the end of October that we will discuss later. Remarkably, the top panel shows that while the followers of @nytimes undergo the synchronization period related to “COVID” topic, those of @FoxNews on the contrary, decrease their similarity, indicating that the “COVID” topic does not act as a synchronizing event for them.

It should not be concluded that exclusive @FoxNews followers at this time do not talk about the #covid topic, but rather that the selection of topics they talk about becomes more inhomogeneous. In fact, we have found that the covid topic is not the most used one of this subset of users (see Supplementary Material) .

The very high peak of the end of October is present in all the curves in Fig. 7, it corresponds to the #endsars topic, mentioned above, and it disappears when the corresponding topic is suppressed from the topic vectors.

The cross-similarity curves between exclusive followers of @FoxNews and a randomized sample of all users is near zero most of the time, and becomes negative around the end of March, where all the other self similarities were increasing. After July and before the the #endsars related peak mentioned above, the cross-similarity approaches zero and so do all the self-similarities curves, with the exception of the @FoxNews exclusive followers. Showing again that those users talk, in general, about the same topics in the same terms, regardless the external events that may drive the attention of other users.

## Discussion

We have studied the dynamics of interactions between the information agenda of a traditional medium, The New York Times, and the discussions that its followers hold on Twitter. We also compare with the discussion held by the followers of other media among the most followed in U.S. involving TV news chains, newspapers, bi-weekly magazines, and press agencies.

Building a semantic network of hashtags with the only assumption that two hashtags used in the same tweet refer to the same subject, we are able to automatically detect the topics discussed in Twitter by community detection in this network. For the NYT journal, the topics are identified by the keywords chosen by the journalists to label their articles.

The entropy of hashtags and keywords usages captures the structural differences among these two kind of media: the curves of the entropy of the vocabulary used by the followers of all the media in Twitter show very similar dynamics including minor details, but all of them show a dynamical behaviour that is different from that of the NYT journal. We observe that the journal is much more concerned with political news than *its own followers*, as shown by the sudden decrease of keyword entropy located around key political dates, for example, during the electoral period. Our results show that the entropy of the vocabulary of the set of @FoxNews followers is significantly lower than for any other media at any time.

Regarding the agenda setting question, a relevant signal is found around the hashtag #Blacklivesmatter, referring to the killing of a black citizen during a police intervention. We show that this discussion was originated on line and was treated by the journal short afterwards.

The analysis of rank diversity of hashtags and keywords uncovers a counter intuitive result: instead of finding the first ranks completely dominated by the few forms of COVID-19 hashtags in Twitter, a high variability of the used hashtags dominates, and only the two first ranks have relatively low variability, which is nevertheless high enough so as to contain hundreds of hasthags. The situation is completely different for the journal, which shows a slowly growing rank diversity of keywords, starting by very low values. This difference is expected as keywords, unlike hashtags, are curated and correspond to the sections of the journal that obey to a hierarchical order. Interestingly, the rank diversity in Twitter is also very different from that observed in Weibo (the Chinese version of Twitter)^52^, which looks more like the rank diversity in the journal where keywords are curated.

The interaction between the journal and its followers has also been explored by studying the patterns observed in the distribution of time delays of direct and indirect responses of the followers, to the articles and tweets posted by the journal. The main observation is the broad spectrum spanned by the time delays of the responses going from seconds up to a week, which may be surprising given the continuous flow of posts in Twitter.

Similar heavy tail behaviour has been identified in studies of the distribution of delays in cascading processes^53,54^, where the models proposed to explain these patterns mainly combine preferential attachment mechanisms with queuing processes^55,56^. However, here we identify a similar distribution of response times in a different setup: instead of following a single cascading effect triggered by an initial seed, which requires for the source tweet to be detected by the users who will potentially retweet (hence the preferential attachment mechanism proposed), we study the behaviour of users who are in principle, automatically exposed to each of the source tweets because they have decided to follow the journal’s account. This questions the pertinence of the preferential attachment hypothesis to explain this observed pattern.

On the contrary, the extremely similar behavior of all direct interactions suggests that this process may strongly be influenced by a queuing process in the users’ timeline, where older tweets might be pushed out quickly by newer tweets. In this case, the first retweets of an article may trigger more retweets from the users that might have lost the original tweet from @nytimes in their timeline, in a manner of a self exciting process^49,50^.

It is not straightforward to foresee a single general hypothesis to explain the heavy tailed shape of the delay times distribution. A detailed analysis conditioned on the section of the NYT in which the articles were published, shows a dependence of the delay times on the sections, suggesting that some types of news have longer lifetimes than others. On the other hand, our analysis of indirect reactions, where users post tweets containing links to articles of the NYT, i.e., by clicking the ‘share via Twitter’ button on the NYT website, shows reaction times that are as expected, much slower.

Finally, the dynamical similarity among groups of users allows to detect that, while most of the users synchronize their discussions around the date of lockdown, a singular behaviour is observed for exclusive followers of @nytimes and of @FoxNews. The similarity of the former, although increasing in this period, is sensitively lower than the similarity of the global population, while for the latter, it shows in this period, the only long lasting decrease of similarity (about a month).

The relatively high and constant values of similarity (except for the large peak related to #endsars) along with the low entropy of the vocabulary of the exclusive followers of @FoxNews strongly suggest that this group constitutes an echo chamber. Moreover the cross-similarity among exclusive users of @nytimes and @FoxNews, is almost always negative (except for the singular #endsars peak), which is an objective measurement of the strong separation of the subjects of interests of these two groups.

## Conclusion

We present a dynamical study of the interactions between a traditional medium, the NYT journal, and its followers in Twitter, and we compare them with the behaviour of Twitter users who follow other media of different kinds (written press, television, and press agencies). It is important to stress that we are not interested in the behaviour of a random sample of Twitter users but we are focusing instead on Twitter users that are interested in news, who could be thought to be a priori more susceptible to media influence.

Our results show that as long as the users follow different media, the similarity among them is almost independent of the media sources they follow. On the contrary, the similarity becomes significantly different when observing sub-populations of *exclusive users*, those who follow one medium account exclusively. We also show that this difference between sub-populations is dominant around the first wave of COVID in the U.S., which in spite of being a public health topic that affects all populations, induces a differential behaviour on sub-populations who exclusively follow different media.

One important feature of our study is the fact that we avoid introducing selection bias by choosing a priori some group of words. Here we keep the whole discussion as it is and we let the topics emerge from the community detection process on the semantic networks.

Finally, we cannot stress enough the importance of choosing different independent quantities to analyse the data: it is the combination of the entropy of vocabulary with the similarity among the users which allows to objectively show the singularity of the exclusive followers of @FoxNews with respect to the baseline population. In the same way, the comparison of the dynamics of entropy, topic evolution, and similarity, shows that although #elections is a hot topic for the journal, the synchronization of its followers around it, although measurable, is relatively lower compared with the #Blacklivesmatter topic. Moreover, in spite of the general difficulty of detecting causality, the comparison of the dynamics of entropy and topic evolution shows that the latter originated on Twitter before being treated by the NYT.

In summary, we present an automatic detection method of discussion topics on social networks, which along with a set of independent measures on the obtained data, brings a lot of information with a minimum of assumptions (here the semantic link among hashtags and among keywords), and should be the entrance gate to more detailed analysis that could focus on the treatment of specific topics or the detailed behaviour of specific groups.

## Methods

In this section we present the data set used in this work, explaining the rationale leading to this particular choice, along with the procedure used for its collection from different data sources. We define the semantic networks built with these data and we explain how we automatically detect the set of topics under discussion and the evolution of the attention each user pays to them.

Moreover we also give the mathematical definition of the observables used to characterize the dynamics of discussion in Twitter and that of the treatment of the news by the NYT over the studied period.

### Data collection

#### Data from Twitter

**Table 2:** Data collected from Twitter. Top panel: random sample of the about 46M followers of the NYT. Bottom panel: followers of the other media described in the text.

@nytimes	Total collected users	8’151’587
	Total collected tweets	502’647’015
	Number of tweets with #	83’237’523
	Number of distinct #	12’937’293
	Number of users quoting/rt/reply	226’630
Other media	Total collected users	1’771’170
	Total collected tweets	96’551’331

We first recall briefly the standard vocabulary used to name different elements of the Twitter micro-blogging platform. Users can engage on many different levels with each other. Each user has a *Twitter handle*, which starts with ‘@’. They can write *tweets*, short messages consisting of up to 280 characters, which may also contain images, videos or sound, and which are shown to their *followers* -other users subscribing to the their accounts- on their *timeline*, the list of latest posted tweets. However, even non-followers can see and interact with them (except for *private tweets* which are not part of our dataset). Users can *retweet* the tweet of another user, which means that they share this tweet with all their followers. They can *quote* a tweet, meaning that they republish the original tweet with a comment. Finally, they can *reply* to a tweet, which starts a discussion connected to the original tweet. Tweets may contain *hashtags*, which are arbitrary strings of characters prefixed by the character ‘#’ , often used to tag the tweet. Tweets can contain a *URL*, which typically links to an external website.

Due to the very large number of followers (about 46 million) of the @nytimes, the official Twitter account of the NYT, we have chosen for this study a random sample of them, according to the following procedure:

- We first obtained the list of the *user ids* of all followers of @nytimes, using the Twitter’s official REST API. This list was collected over a few days in the last week of June 2020.
- We randomized the obtained list.
- On July 1st 2020, we requested up to the last 3200 tweets (this number is a limitation of the Twitter API) of a sample of about 8M of these accounts.
- Roughly every 2 months we requested, for all users in our sample for which we already found tweets for the year 2020, the new tweets they published since our last query.

Table 2 gives the main characteristics of the data used for this study.

At the beginning of March 2021, we had collected up to half a billion tweets published by more than 8M (8,151,587) followers.

As it is well known that only a minority of Twitter users include their geolocalization in their profile, we have chosen not to control for this variable so as to avoid artificially diminishing the number of collected users. However, since the US is the largest market both for Twitter and NYT, we expect that most followers are indeed located in US. As a consequence, although we cannot rule out that the dataset contains tweets of users living abroad, we will naturally focus on events that are relevant to the US in order to tag the chronology of the study. The pertinence of this choice is supported by the fact that topics which are popular in the US are dominating the discussion, and we show that it is possible to identify the rare exceptions.

This dataset, in the form of user and tweet ids, is available at^57^.

Although our method enables us to collect a large sampling of a specific subpopulation of Twitter, avoiding biases that may be introduced by filtering, for example by hashtags, we discuss below some limitations that might still remain in this data set, along with an estimation of their potential influence in our study.

- Due to the limit set by the API (it delivers only the last 3200 Tweets of the requested user), we risk to systematically miss tweets of very active accounts: those who would have tweeted more than 3200 tweets between January 1st and July 1st or those who would have exceeded that limit during the $\sim 2$ month period of each collection step hold after July 1st 2020 until the end. Although most of such accounts are automated (*bots*) or institutional ones, like @nytimes itself, one cannot rule out a priori the existence of accounts of very active individuals. Notice that such users need to write at least about 18 tweets per day, on average, in the first six months and many more in the following collection periods (every two months), which is certainly possible but not typical of the standard user. Nevertheless, in order to evaluate to what extent our sample is likely to contain *incomplete users* -accounts for which we could not get the full set of the content they published- we set a conservative criterion to detect them. We count the number of users for which we collected more than 3000 tweets. This strict bound leaves a generous room for deleted tweets, which although not downloaded, still count against the 3200 limit. Since we can collect at most 3200 tweets at each point in time, we cannot exclude a priori, that a user wrote all these tweets and even more during one of our collections cycles (and few or none in the other cycles). However, we do not observe such inhomogeneous behavior, in spite of the fact that our sample contains users who exceed 18000 tweets in all the period. We are therefore confident that the strict bound set here overestimates the fraction of incomplete users considerably. According to this strict criterion, we estimate that only less than $0.4\%$ of all accounts are incomplete. Thus, the potential error should be small, in particular considering that our study makes a stronger usage of the number of unique users rather than the number of tweets.
- The list of followers was fixed at the beginning of the study, such that we do not include users which started following @nytimes after July 1st 2020, slightly underestimating the influence of new and short lived accounts.
- In the same way we cannot exclude that some accounts we sampled stopped following @nytimes at some point during our period of study.
- Naturally, we do not consider in our sample tweets from deleted, suspended and private accounts.

Following a similar technique, we also collected a smaller sample of about a million users who do not follow @nytimes but who follow at least one of other seven most followed US news media accounts. We do not include followers of secondary accounts (e.g., those of “breaking news”, like @CNNbreaking).

Table 3 describes the different sources from where we have collected the sample of Twitter users interested in US news that we have studied in this work.

**Table 3:** Number of followers of the Twitter accounts of the studied media.

Name	Media type	Followers
CNN	TV news	53,242,242
FoxNews	TV news	20,121,721
Reuters	news agency	23,238,148
Associated Press	news agency	15,127,593
TIME	bi-weekly magazine	18,065,949
Wall Street Journal	newspaper	18,705,760
The Washington Post	newspaper	17,791,609
The New York Times	newspaper	46,808,154

We collected a uniform sample of these users proportional to the number of followers each medium has, in the last weeks of March 2021. This means that the problem of missing tweets from very active accounts is worse for this data set. However, the fraction of incomplete accounts remains small $< 0.3\%$ (even smaller than for the @nytimes dataset, because we only had one cycle causing fewer false positives). Again, this dataset in the form of user and tweet ids is available at^58^.

Finally, we also collected all tweets of the @nytimes account for the period, referenced by retweets, quotes or replies of their followers.

In this study we only use metadata of the tweets: hashtags normalized to lower case (i.e., we treat #covid-19 and #COVID-19 as the same hashtag) and URLs. We do not extract further data from the remainder of the tweet, neither text nor images nor videos. Nevertheless, we will show that this minimalist information contained in the tweets already provides a rich image of the public discussion in the platform.

#### Data from the NYT

Table 4 describes the main figures involved in the analysis of the publications of the NYT journal during the same period.

**Table 4:** Data concerning the publications of the NYT journal in the considered period.

Number of articles	62,138
Number of tweets posted by @nytimes	33,446
Links to articles in @nytimes tweets	20,496
Number of distinct keywords	45,016

In addition to the data from Twitter users, we collected the metadata of all articles published by the NYT either in print or online using their *archive API*. This dataset includes in particular, a set of keywords for each article, which lists subjects, persons and locations referred to in the article. Moreover, it provides unique identifiers, which we used to connect URLs encountered in tweets, to a NYT article, an otherwise non trivial task, since an article can have multiple valid URLs.

The dataset that indicates which tweets link to which articles is also available at^57^.

### Observables

We detail in this section the quantities or *observables* that we used in this study.

#### Entropy

The entropy of the hashtag distribution over a timeframe *t* is defined as:

1 $$\begin{aligned} S_t = -\sum _i p_t(i) \ln p_t(i). \end{aligned}$$

where $p_t(i)$ is the probability distribution calculated as the ratio between the number of unique users that have used hashtag *i* and the number of different pairs (hashtag, user) within the time frame *t*. By considering unique users we diminish the influence of very active accounts (e.g., spammers). We calculate the entropy daily with a rolling time frame of seven days to remove the well known influence of the lower activity on weekends.

#### Topic detection

In this study we are interested in comparing the dynamics of subjects published by a traditional medium, like the NYT, where professionals choose the information to be issued, with the dynamics of discussion that its followers hold on the Twitter platform. To do so one needs to identify the *topics* that are discussed in both media. The literature on topic modeling is quite extense, and several unsupervised models exist that can extract topics from textual corpora, either based on semantic network analysis and/or topic modeling^59,60^.

In Twitter we can adopt hashtags, which are used to tag the messages, as a proxy for the subject of the tweet. However, multiple hashtags may address the same topic. A common strategy to follow the discussion about a topic is to pre-select the hashtags that are supposed to be related to the topic. Here we use a different approach where the topics emerge from a *semantic network* of hashtags^37,61^. The vertices of this network are the hashtags found in our dataset, and the weighted edge between two nodes represents the number of different users that used those hashtags together in at least one of their tweets. In clear, if the same user publishes many tweets including the same pair of hashtags, it contributes to the weight only once. The rationale behind this construction is that two hashtags used in the same tweet refer to the same subject; in fact, previous work has shown that hashtag co-occurrences in tweets are mostly coupled with semantic relations^62^. Finally, in order to avoid spurious relationships we set a threshold for the link to be meaningful and we prune all edges whose weight is below 10. In this way, hashtags talking about the same topic should be strongly connected and synonymous hashtags, which only seldom appear in the same tweet, should be strongly connected to the same common nodes.

By performing community detection on the semantic network, we detect the groups of hashtags that are more tightly connected among them than with the rest^63^. We identify each community with a topic of discussion in the platform.

This topic-community identification may suffer from some ambiguities because some hashtags can belong to multiple topics. For example, if we use OSLOM2^64^ for community detection, which allows for community overlap, we find that #covid19 which influences most aspects of life, is associated with more than 10 communities. Since overlapping communities are hard to interpret, we finally chose a community detection algorithm, *Infomap*^65^, which provides a disjoint partition. In this case #covid19 will be assigned to one topic. To illustrate the density of this network, a small fraction of it (the induced subgraphs of $\approx 1.5\%$ of the most co-used hashtags) is represented in Fig. S5 of the Supplementary Material.

For keywords obtained from NYT articles, we do not need to perform such a topic analysis, since they are manually curated to already describe topics.

#### Rank diversity

The *rank* *r* of an entity (here either hashtags or keywords) is its position in the list of all entities occurring within a time period sorted by decreasing number of usages. Following^39,40,52^, we define the *rank diversity* *d*(*r*) over a time frame $\Delta$ with a time resolution $\delta$ as the number of of different entities occupying rank *r* over the $k = \Delta / \delta$ time spans normalized by *k*. It therefore can assume values in [1/*k*, 1], where 1/*k* signals that only a single entity was observed on the corresponding rank and 1 that the entity changed for every period. Here, we study the $\Delta = 366$ days of 2020 and use a resolution of $\delta = 1$ day (starting at 0:00 UTC).

This measures how consistent topics of interests are. Low values signal little fluctuations in the importance of the entities, while high values suggest high fluctuation. If *d*(*r*) increases with the rank *r*, it signals that the really important topics are more consistent than minor topics. A decrease could happen if the entities are artificially curated, e.g., limited to a certain number.

#### User similarity

To study how similar users are in regard to the interest they pay to different topics, we applied the method used in^61^. We describe the interests of each user *i* by means of a user description vector $\varvec{d_i}$ of dimension $N_T$, the number of topics (communities) found, which informs about the topic preferences of user *i*.

This description vector is computed in the following way:

1. We build a user-topic matrix, *U*, where each element, $u_{ij}$, gives the absolute number of times that user *i* has used a hashtag that belongs to the community identified as topic *j*.
2. We compute the global topic vector ${\varvec{T}}=\sum _i ^{N}{\varvec{u_i}}$, where $\varvec{u_i}$ is the *i*-th row vector in the user-topic matrix, and *N* the size of the population. This vector gives the total number of times that each topic has been used by all the users in the dataset.
3. We define the vector $\varvec{v_i}$ which gives the difference between the frequency of usage of the topic by user *i* and its global frequency of usage in the population.
  2 $$\begin{aligned} \varvec{v_i} = \frac{\varvec{u_i}}{||\varvec{u_i}||_1} - \frac{{\varvec{T}}}{||{\varvec{T}}||_1} . \end{aligned}$$
  Here the norm $||.||_1$ must be understood as the sum over all the components in the space of dimension $N_T$. The vectors of Eq. (2) thus inform about whether user *i* has addressed each of the identified topics more or less than on average.
4. As we are only interested in the orientation of the description vectors, they are normalized as:
  3 $$\begin{aligned} \varvec{d_i} = \frac{\varvec{v_i}}{||\varvec{v_i}||_2} , \end{aligned}$$
  where $||\varvec{v_i}||_2$ is the standard euclidean norm in the topic hyperspace of dimension $N_T$.

Then, in order to track the evolution of the users’ interests we apply the aforementioned procedure to sliding time windows of 7 days, thus producing a series of matrices $U_t$, one for each day. We shall call $\varvec{d_i}^t$ the description vector for user *i* at discrete time *t*.

We define the similarity between a pair of users *i* and *j* as the cosine similarity between the corresponding description vectors. As the latter are normalized, the similarity reduces to the inner product:

4 $$\begin{aligned} s(i,j) = \langle \varvec{d_i}, \varvec{d_j} \rangle . \end{aligned}$$

We also define the average description vector of a group of users *G*, of cardinality |*G*|:

5 $$\begin{aligned} \varvec{D_G} = \frac{\sum _{i \in G} \varvec{d_i}}{|G|} . \end{aligned}$$

Now we can introduce two indices measuring *collective similarities*:

- The *cohesion* of a group of users, *intra-group similarity* or *self-similarity*, *s*(*G*, *G*), defined as the average similarity between all its users, and computed in the following way:
  6 $$\begin{aligned} s(G,G)&= \frac{\sum _{i,j \in G} {s(i,j)}}{|G|^2} = \frac{\sum _{i \in G}{\langle \varvec{d_i}, \varvec{D_G} \rangle }}{|G|} \end{aligned}$$
  7 $$\begin{aligned}&={\langle \varvec{D_G}, \varvec{D_G} \rangle }=||\varvec{D_G}||^2 , \end{aligned}$$
- The *cross-group similarity* is the average similarity between members of different groups $G_1$ and $G_2$, namely $s(G_1, G_2)$:
  8 $$\begin{aligned} s(G_1,G_2)&= \frac{\sum _{i\in G_1,j \in G_2} {s(i,j)}}{|G_1|\cdot |G_2|} \end{aligned}$$
  9 $$\begin{aligned}&= \frac{\sum _{i \in G_1}{\langle \varvec{d_i}, \varvec{D_{G_2}} \rangle }}{|G_1|}={\langle \varvec{D_{G_1}}, \varvec{D_{G_2}} \rangle } . \end{aligned}$$

## Supplementary Information

Supplementary Figure S1.

## Data Availability

Dataset of user and tweet ids of followers of @nytimes is available at: 10.5281/zenodo.4736651. Dataset of user and tweet ids of followers of other news media is available at: 10.5281/zenodo.4736816.
