# Supplementary material for: Understanding who talks about what: comparison between the information treatment in traditional media and online discussions
Source: Sci Rep. 2023 Mar 7;13:3809. doi: 10.1038/s41598-023-30367-8 (PMC9990029; doi:10.1038/s41598-023-30367-8)
Supplement: Supplementary file 1 — Supplementary Figure S1. [file 41598_2023_30367_MOESM1_ESM.pdf]

Supplementary Material
Understanding who talks about what: comparison between the information treatment in traditional media and online discussions.

Hendrik Schawe,[1,*] Mariano Gaston Beiró,[2,3,†] J. Ignacio Alvarez-Hamelin,[2,3,‡] Dimitris Kotzinos,[4,§] and Laura Hernández[1,¶]

[1]*Laboratoire de Physique Théorique et Modélisation, UMR-8089 CNRS, CY Cergy Paris Université, France*
[2]*Universidad de Buenos Aires. Facultad de Ingeniería, Paseo Colón 850, C1063ACV Buenos Aires, Argentina*
[3]*CONICET, Universidad de Buenos Aires, INTECIN, Argentina*
[4]*ETIS UMR 8051 CY Cergy Paris Université, ENSEA, CNRS, France*

CONTENTS

I. Reaction times 1

II. Similarities 1

III. Main topics discussed by @FoxNews followers 3

IV. Top-10 hashtags of the main topics 5

V. Network visualization 6

VI. List of hashtags in the Covid-19 topic 7

References 10

I. REACTION TIMES

In Figure S1 we present reaction time distributions in general –i.e. adding all the journal sections– (upper-left panel) and for specific journal sections that were not included in Section II.C of the main text. These distributions confirm that the direct reactions – through the website/App "Share on Twitter" link– (purple) are qualitatively diverse from those indirect ones arising in Twitter (as retweeting, replying, or quoting a NYT tweet). These results imply that the Twitter timeline is much more ephemeral as compared to the NYT App or website.

Among the remaining actions, replying and quoting have similar distributions in every newspaper section, and have a minimum delay time close to 10 seconds. Instead, the retweeting delay distributions (green) tend to present an inmmediate peak just after the NYT tweet is published, then falling, and raising again.

II. SIMILARITIES

The sharp peak observed in all similarity curves of Fig.7 of the manuscript correspond to the #endsars topic as can be corroborated in Fig. S2, where the similarities were recalculated after suppressing the usages of the #endsars topic from the topic vectors. This topic makes reference to a demonstration against police violence sparked by videos showing brutality of the Nigerian police organization SARS (Special Anti-Robbery Squad,not to be mistaken for SARS-COVID).

* Hendrik.Schawe@gmail.com
† mbeiro@fi.uba.ar
‡ ihameli@cnet.fi.uba.ar
§ dimitrios.kotzinos@cyu.fr
¶ laura.hernandez@cyu.fr

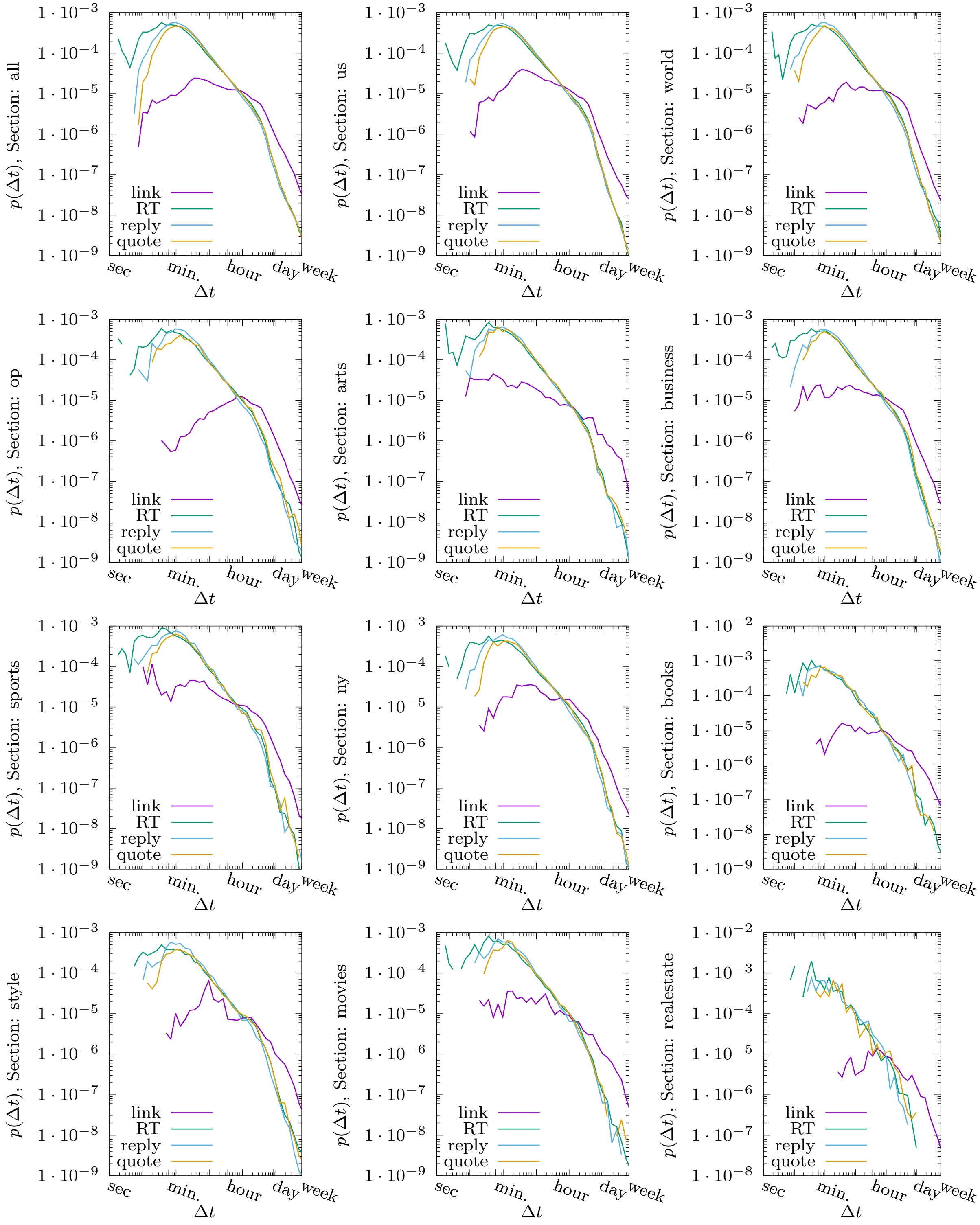

FIG. S1: Delay times Δt between @nytimes posting a link to one of their articles and reactions of their followers. The dark line labeled *link* tracks the delay between publication of an article on the website and the appearance of tweets from @nytimes followers containing a link to it. We show this measurement conditioned on the section of the NYT in which the corresponding articles appeared.

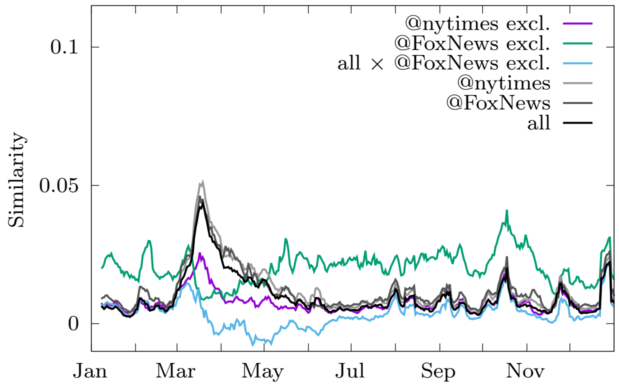

FIG. S2: Dynamics of self- and cross-similarities of different subpopulations corresponding to the followers of different media accounts in Twitter, recomputed after removing the #endsars topic from the topic vectors. In this way we identify the origin of the very high peak in October 2020 shown in Fig.7 of the main text. For clarity we concentrate on the curves involving the followers of @nytimes and @FoxNews, along with a randomized sample, that gathers followers of all media together (labelled "all"). The labels '@nytimes excl.' and '@FoxNews excl.' refer to the subpopulations that only follow the cited media. 'all × @FoxNews excl.' is the cross similarity between the exclusive followers of @FoxNews in our dataset and all users (including the followers of @FoxNews) in our dataset.

This peak is caused by many hashtag usages by a relative low number of users, such that it does not appear in the entropy which only considers usages by unique users, stressing the importance to analyse data using different indicators. For comparison, each user, who used #endsars, did so on average 14.1 times, while each user who tweeted #blacklivesmatter, did so on average only 3.3 times, such that the latter has a much broader support, and therefore causes a stronger signal in Fig.1 of the main text.

On the contrary, the similarity takes into account the number of usages of a topic by each user. Therefore those using #endsars have the corresponding component of their topic vector, much larger than the others, such that it is almost aligned with the #endsars direction, giving rise to the strong increase in similarity. We could identify that more than $\approx 40\%$ of the #endsars hashtags were tweeted by users, who specified "Nigeria" as (part of) their location. Since most users do not specify any location, we conjecture that the hashtag was mainly used by users in Nigeria, who were captured because they follow the most popular media in U.S.

In Figure S3 we present the dynamics of the self-similarities for exclusive (green) and non-exclusive (violet) followers of the different news agencies.

The behaviour of self-similarities of exclusive followers depend on the media they follow. For the exclusive followers of @TIME, @FoxNews and @WSJ the self-similarity tends to be higher than for their general followers, with the exception of the beginning of the COVID-19 pandemics, between March and June.

On the contrary, from June onwards, the dynamics of @nytimes, @CNN, @AP and @washingtonpost exclusive and non-exclusive followers are quite similar within each media, showing also similar peaks.

An interesting peak is also observed at the beginning of February for exclusive followers of @AP and @FoxNews, a signal that is absent of the similarities of the exclusive followers of other media.

III. MAIN TOPICS DISCUSSED BY @FOXNEWS FOLLOWERS

In order to complement Fig. 2 in the main text showing the dynamics of the largest 8 topics discussed by the @nytimes followers, Fig. S4 shows the analogous time dynamics of the main topics discussed by exclusive and non-exclusive @FoxNews followers in Twitter.

The most active topic for @FoxNews followers is related to the presidential elections, while the coronavirus pandemics is at a second place, as opposed to the behavior of @nytimes followers in Fig. 2 of the main text.

The treatment of Black Lives Matter is also fundamentally different between @nytimes and @FoxNews followers, being the most discussed topics among the former during its peak, and being strongly associated to "content marketing strategies" among the latter.

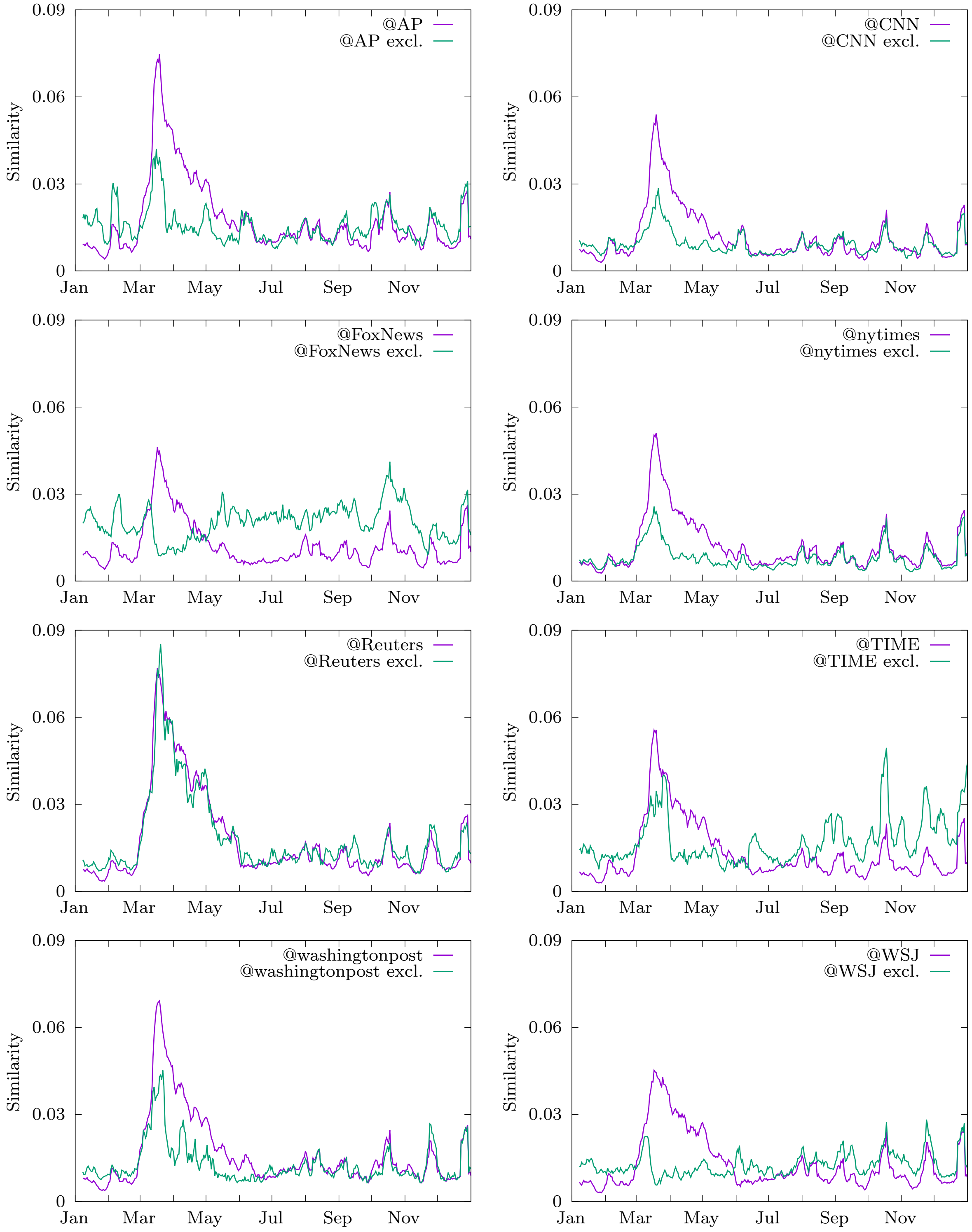

FIG. S3: Self-similarities conditioned on which media outlet the users are following (excluding the #endsars topic).

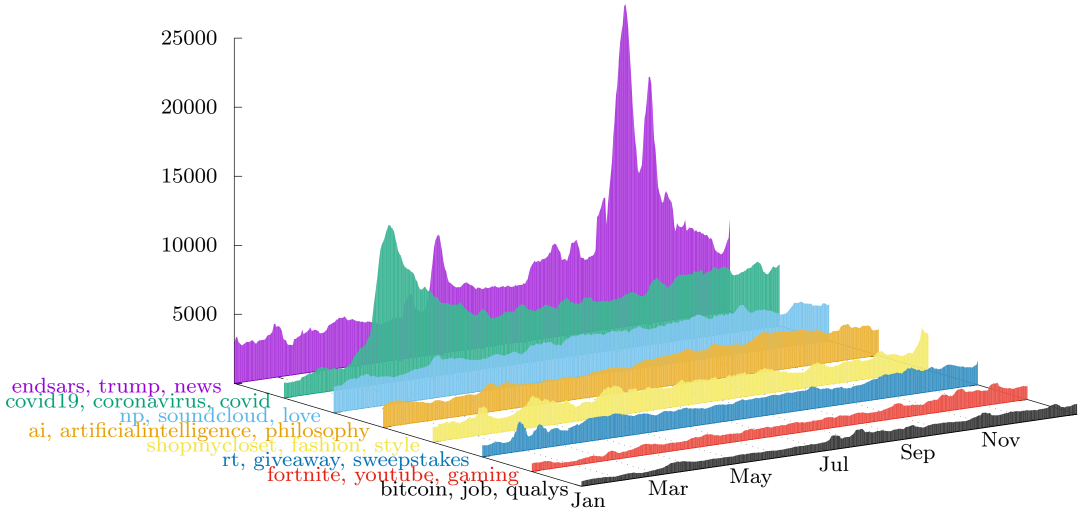

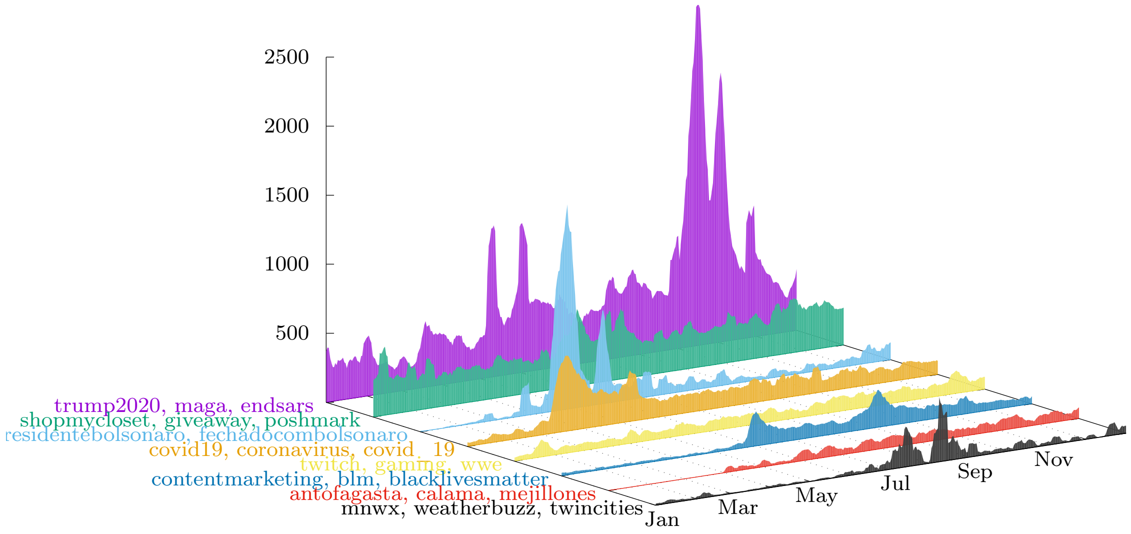

FIG. S4: Topic dynamics for @FoxNews followers (top) and exclusive @FoxNews followers –i.e. not following any other captured media– (bottom).

IV. TOP-10 HASHTAGS OF THE MAIN TOPICS

Table S1 lists the main hashtags associated to the most frequent topics discussed by the NYT followers. While, as expected, the most used topic over the period is the one related to COVID-19 pandemics, other important topics follow important events of the period like the *BlackLivesMatter* protests and the US elections. Notice that other

Topic 1	Topic 2	Topic 3	Topic 4	Topic 5
covid19	newprofilepic	blacklivesmatter	usa	love
coronavirus	stayhome	georgefloyd	maga	respect
covid	stayathome	breonnataylor	truth	life
covid_19	staysafe	justiceforgeorgefloyd	america	motivation
covid-19	zoom	defundthepolice	justice	inspiration
covid2019	stayhomestaysafe	blacklivesmattters	breaking	gratitude
corona	safety	policebrutality	media	success
pandemic	flashbackfriday	icantbreathe	breakingnews	humanity
coronaviruspandemic	caresact	protests2020	maga2020	power
coronavirusoutbreak	celebration	georgefloydprotests	uselection2020	positivevibes
**Topic 6**	**Topic 7**	**Topic 8**	**Topic 9**	**Topic 10**
trump	facebook	vote	wearamask	superbowl
biden	florida	sundayvibes	nature	nfl
gop	newyork	sundaythoughts	blessed	superbowlliv
scotus	california	election	sunset	snl
2020election	texas	saturdaythoughts	tgif	chiefskingdom
electionresults2020	london	fraud	stayhealthy	billsmafia
trumphascovid	whatsapp	saturdayvibes	amazing	browns
bidenharristosaveamerica	paris	stopthesteal	staysafestayhome	chiefs
republicans	reading	draintheswamp	staystrong	nflplayoffs
whitehouse	losangeles	democrat	sunrise	nfldraft

TABLE S1: Top hashtags present in the most common topics.

hashtags evoking the pandemic (and also elections) are part of different topics. This is related to the fact that both the pandemic and the elections intervene in several aspects of public discussion and the method is able to detect so.

V. NETWORK VISUALIZATION

In Figure S5 we offer a visualization of part of the semantic network of hashtags, including the 1.5% most frequent hashtag pairs. Here, the size of a node (hashtag) represents the number of hashtags that it is linked to. Colors represent the community structure found by Infomap [1].

#covid and #coronavirus, in the pink community, stand out as the most connected hashtags in the network. Other discussion topics are related to the elections (gray), cryptocurrencies (violet) and technology (light blue).

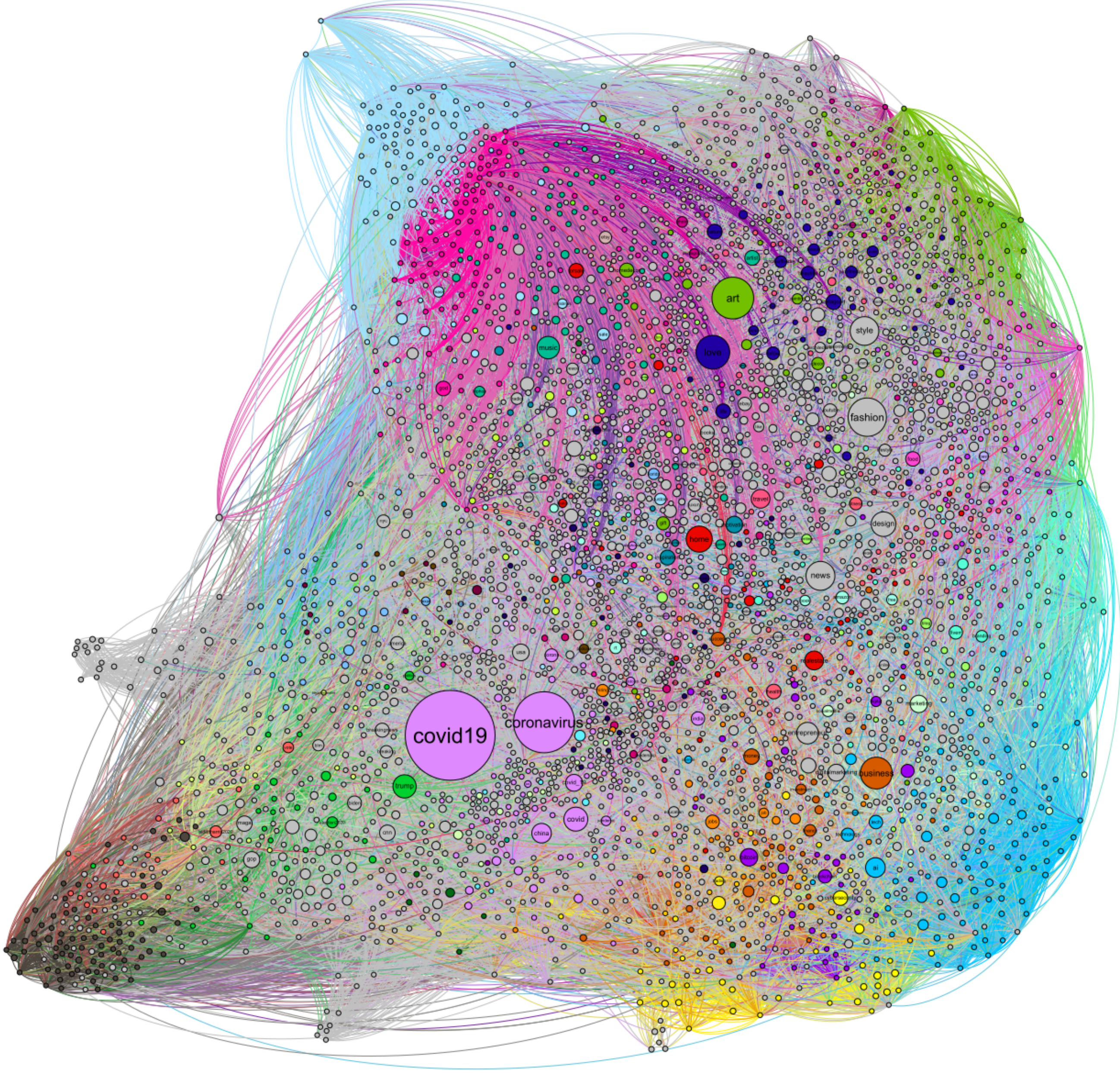

FIG. S5: Visualization of a part of the semantic hashtag co-occurrence network ($\approx 1.5\%$ of the most co-used hashtags are visualized) in a force-directed layout. The node size represents the total number of co-usages, the colors distinguish the communities detected by Infomap. The labels are hashtags and omit the leading '#'.

VI. LIST OF HASHTAGS IN THE COVID-19 TOPIC

covid19	coronavirus	covid	covid$_1$9
covid-19	covid2019	corona	pandemic
coronaviruspandemic	coronavirusoutbreak	healthcare	vaccine
coronavirusupdate	cuarentena	covid19pandemic	pandemia
coronavirusupdates	virus	masks	mask
nhs	coronaviruslockdown	covid19uk	wuhan
coronavirususa	publichealth	coronacrisis	quedateentucasa
covididiots	cdc	coronavirusuk	facemask

coronaupdate	sarscov2	vaccination	staythefhome
medicine	hospital	coronaviruspakistan	flu
herdimmunity	physicaldistancing	restezchezvous	covid19italia
trumppressbriefing	covid19usa	skincare	commonsense
fase2	hospitals	coronavaccine	costco
coronaupdatesinindia	pressconference	fda	coronainpakistan
covid19vic	denmark	coronavirusvaccine	covidtesting
drjillbiden	covid19ireland	pressbriefing	covidindia
influenza	coronavirusbrasil	vaccin	rural
coronapandemic	immunity	contagion	coronavirusfrance
humans	coronastopkarona	estadodealarma	covidactnow
trumpcrash	coronavirusnobrasil	chine	cyprus
2019ncov	alemania	disease	hygiene
herdmentality	lockdownpakistan	coviduk	santé
vitamind	covid20	viruschino	icu
operationwarpspeed	madagascar	level5	smoking
toolittletoolate	plasma	immunesystem	coronavirusitalla
estadodeemergencia	genetics	1a	desescalada
crispr	hydroxycloroquine	europeday	2020sofar
londres	sindhgovt	italie	covid19nl
ventilator	communityshield	infection	panamá
coronavirusireland	responsabilidad	coronamaatregelen	coronaviruske
coronvirusitalia	airtravel	historymatters	preexistingconditions
covid19ecuador	epidemiology	fatigue	reopeningschools
bats	coronaviruscrisis	vaccinated	coronavirusenargentina
coronavirusfacts	antibodies	persconferentie	viruses
germania	veneto	pandemiamundial	impfstoff
asymptomatic	pneumonia	mediavirus	b117
genomics	sanidad	antibody	ambulance
mutation	californialockdown	neurology	novelcoronavirus
westandtogether	ncov	vaccins	pharmaceuticals
temperature	h1n1	immunotherapy	coronavirusseattle
epitwitter	covid19testing	osaka	transmission
covid19vaccines	coronachainscare	wuhanpneumonia	getusppe
cardiovascular	vaccinocovid	diamondprincess	algérie
howwefeel	inflammation	fever	clinicaltrial
covid19peru	notmykid	mañanera	foreverychild
cancelthedebt	schengen	hhs	uzbekistan
cv19	rivm	immunology	coronavirusstrain
restateacasa	windsor	andalucía	coronavirusespaña
schulen	mortality	stemcells	solidaritecovid19
wineshops	bloodpressure	infectiousdiseases	corvid19uk
respiratory	ripartenza	sepsis	prevent
rna	crowdsourcing	corinavirus	coronavirusplantao
telemedicina	mitigation	cough	pil
genetic	lungs	criticalcare	laboratory
n95mask	bioinformatics	curevac	virology
covid19quarantine	pressercovid19	pathology	covid19impact
antibioticresistance	cov	2ndwave	zika
peston	virtualtours	portaaporta	antiviral
shinzoabe	bioethics	cholesterol	tier2
cell	mileg	genome	adolescents
convalescentplasma	venceremos	pv	bcg
migrantsday	diagnostic	pascua	infectioncontrol
gene	covid$_1 9ireland$	segundaola	genes
newsmedia	immunization	contamination	wearetogether
aislamientosocial	anaheim	covid19seattle	pruebas
mv	lorraine	herb	impfungen
azithromycin	futureofeurope	idtwitter	multilateralismmatters
grandprincess	antibiotic	ards	talsagharreybehew
mutations	postgraduate	coronaviruspy	travelnews

ddj	microscopy	diarrhea	pekin
usp	trumpregime	covid19dz	rapidtest
lockdownzim	transcription	typhoidmary	euros
mylifematters	mélenchon	kashmircovid19	coronaheroes
manufacture	kontaktverbot	amlomientenohaytrabajo	cardiovasculardisease
kawasakidisease	ruralbroadband	compound	anosmia
mutant	neurons	covid19cmr	everydaycounts
alarma	herdenimmunität	bioengineering	iwmd20
nanoparticles	biomarker	antioxidant	herdimmunityismassmurder
luzonlockdown	opensafely	rikers	genomic
trumpisresponsible	buildforwardbetter	vraioufake	antigen
punefightscorona	teatri	coronavirusjapan	ace2
zoonosis	intensivecare	beautycare	coronavirusalgerie
aceh	biologicalwarfare	protegeteyprotegealosdemas	cremona
wuhanlockdown	prenezsoindevous	bilan	virusoutbreak
tnfightscorona	molecularbiology	africaresponds	covid19japan
efficacy	grandest	johnshopkinsuniversity	serology
thrombosis	protectallpeople	commoncold	biopharmaceutical
pathtwitter	fightthevirus	safewater	fvg
europeansagainstcovid19	coughing	organoids	checkingin
dependencia	limassol	coronavirusafghanistan	trumpfakedcovid
maternaljustice	lungdisease	hypoxia	stopthesurge
stareacasa	pasalavoz	pathogen	estonian
proteomics	cjrs	enzyme	hormone
autophagy	theshield	jemefaisvacciner	sneezing
diamondprincesscruise	hipertensión	democrathoax	acr20
rosja	whwweek	lopinavir	cd4
estrogen	preventative	cytokine	cdnimm
clothmask	coronamask	weneedwho	d614g
moselle	invitro	piensaantesdecompartir	hie
placenta	acip	theeagle	molecule
frictionless	lipids	tshamakaya	peptides
solidarityinaction	contracosta	sarcov2	coronavirusdisease
covid19catalunya	pandemien	bayareacoronavirus	crisiscovid19
bloodplasma	coronavirusfree	fluorescence	besomebody
stayhometo	ritonavir	mnch	protegetedelcovid19
neutrophils	yonoolvido	healthyreturns	cytokines
immuneresponse	travelbannow	adenovirus	standagainstcorona
trumplawsuits	dices	eular2020	creatorsrespond
preclinical	noveastvazteca	protectlives	pathophysiology
compassioninaction	dia31	clasepolitica	drugrepurposing
pkv	solidwastemanagement	kn95mask	schwereschuld
transcriptomics	apoptosis	memlekethareketi	heparin
washoecounty	mesoamérica	restez	covidfood
pelotas	shivajinagar	spikeprotein	druckerforum
viruschinês	rethinkjails	macrophage	receptor
vaccinequity	primarycarecovidchat	protegezvous	insleemustgo
phylogeny	ampath20	proteome	assay
corticosteroid	nasalspray	risingtothechallenge	kn95masks
aceinhibitors	geneexpression	kf94	imaginarium
mouthmask	aminoacid	autoantibodies	nanobodies
covid19ark	eltiempochv	managingthefutureofwork	davidabel
eulocal	coronatravel	covid19vaccin	cbalive
glnrtoday	healthcarespending	bcell	koronavirüstürkiye
monoclonalantibody	businessresponseirl	covidcrimewatch	itexit
immunohistochemistry	immunoglobulin	centralnervoussystem	kinase
usofanxiety2020	district8	glycans	spectrometry
fixmygop	anticoagulant	ruralhousing	türkiyekazanacak
thedosecbc	aarpminute	japandemic	covidunder19
fearlessscience	eubef20	méxicocontigo	contagiavoluntad
rosji	scngoncv	restonsvigilants	nucleicacid

testday	lymphocyte	iowansunite	getamericabacktowork
genetictest	fomite	angiotensin	covidcruiseship
covidjapan	paltc	mejorprevenir	markenkommunikation
adbnews	estradiol	tolonewsbreak	lonconf20
coronavirusrestrictions	pseudovirus	cytometry	glycoprotein
680news	casefatalityrate	immunoassay	coronavirusrta
covid19maine	glycan	ocda	covidcorps
paltc20	heritageinlockdown	realtalkwithroshini	westvalleycity
umichinthenews	400000dead	mexicoseeyousoon	diseasemodeling
polymerase	bakisalama	laliga2020	medicinalaboral
findelsistema	imstayingathome	hkinsights	calibrescientific
abcpm	tecuidasnoscuidas	capsid	covid19l
hlbinsights	sprotein	genomicsequencing	ddimer
nucleotide	eccunion	debatesdigital	recogurus
9newsat6	cysteine	livewiththevirus	chemokines
eastto	chemokine	adbimpact	severeacuterespiratorysyndrome
untalks	covidneo	shccares	bullshitwatch
consuladosmxcovid19	iwnews	williamskastner	insurancequestionsanswered
codon	triconinfotech	urbeyorbe	istandwithchriskrebs
5thingstoknow	insightout	immunitiy	usnhospitalheros
unkor	milagrow	impactmanager	securethecall
marianotecuida	dobbydesantis	dishonestdiscourse	gumc
affiliatesunidos	oursweepersourheroes	reproductionnumber	mcmillanllp
cvhaction	peopleareoureconomy	annalsoncall	sdgtalks
ruralconversations	shutdownaznow	fuvmagazine	euromunicipalismo
sosestudiantesuniversitarios	thefedstories	blgpublication	japanfacts
monocyte	microarray	askacluva	mlbei
díadepruebas	alertaencárceles	ggiilluminations	paltclead
estamosprontos	whatswrongwithwisconsin	yoencasaestoyseguro	nychbleaders
mosstakes	medicalcountermeasures	nhstechhero	yotengoelcoronavirus
includenurses	díadeprueba	togetherweendcovid19	centrocívicomexicano
livewellhealthy	japanemic	thefutureofeducationsummit	japanfact
acanadian	covid19infographic	srcori	sportedgeug
coronavirustheories	unitedstatescoronavirus	sabkaramazan	cuarentenaespaña
baatonbaatonmeinsehat	balancevenezuela	resiliencewebcast	quosalud
quociencia	pruebasincosto	coronavirusinfographic	bozzer
balancenacional	disabilityassessor	dorchestercountysc	intergremialpresente

TABLE S2: Hashtags found in the community associated to COVID-19, ordered by decreasing usage.

[1] M. Rosvall and C. T. Bergstrom, Maps of random walks on complex networks reveal community structure, Proceedings of the Na- tional Academy of Sciences 105, 1118 (2008), https://www.pnas.org/content/105/4/1118.full.pdf.
